# Supplementary material for: Study of Arbitrarily Low Shear Rate Rheology Using Dissipative Particle Dynamics
Source: J Chem Theory Comput. 2026 Apr 8;22(8):3779–95. doi: 10.1021/acs.jctc.5c01825 (PMC13130868; doi:10.1021/acs.jctc.5c01825)
Supplement: Supplementary file 1 [file ct5c01825_si_001.pdf]

Supporting Information for: Study of arbitrarily low shear rate rheology using dissipative particle dynamics

Francesco De Roma,† Luca Maffioli,‡ Edward R. Smith,*,¶ and Antonio Buffo*,†

†*DISAT, Institute of Chemical Engineering, Politecnico di Torino, C.so Duca degli Abruzzi 24, Torino 10129, Italy*

‡*Department of Mathematics, School of Science, Computing and Engineering Technologies, Swinburne University of Technology, P.O. Box 218, Hawthorn 3122, Victoria, Australia*

¶*Department of Mechanical and Aerospace Engineering, Brunel University London, Uxbridge UB8 3PH, UK*

E-mail: edward.smith@brunel.ac.uk; antonio.buffo@polito.it

1 DAV–TTCF discrepancy and SLLOD implementation in LAMMPS

As discussed in the manuscript, the discrepancy observed in Fig. 2 and Fig. 6 is attributed to the current implementation of SLLOD and the Lagrangian rhomboid boundary conditions (LRBC) in LAMMPS (version 29Aug2024). These issues are analyzed in the recent preprint by Sanderson and Searles [1], which also provides a modified LAMMPS version for testing[1]. Sanderson and Searles [1] reported that subtle non-reversibility and/or imperfect energy conservation in commonly used SLLOD integration schemes can produce a systematic bias in

[1] https://github.com/ssande7/lammps/commit/0a580187681f71f32260905ff8c67bf6ba896364

the direct ensemble average of the pressure tensor, leading to an apparent viscosity error. Therefore, the modified LAMMPS version was tested here. For the LJ–WCA fluid, results at $\dot{\gamma} = 10^{-6}$ are reported in Figure S1. The modified implementation yields a clear improvement in the agreement between TTCF and DAV, most evident at short and intermediate times (Figure S1b). At longer times, the DAV estimate becomes dominated by noise, which makes a quantitative comparison less informative.

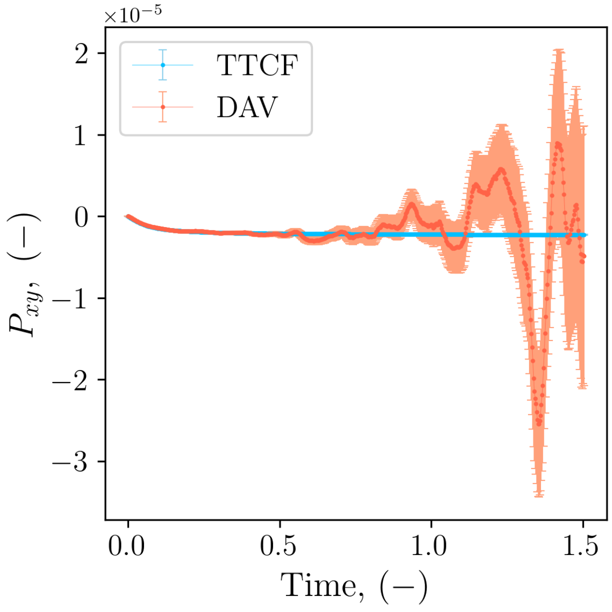

(a)

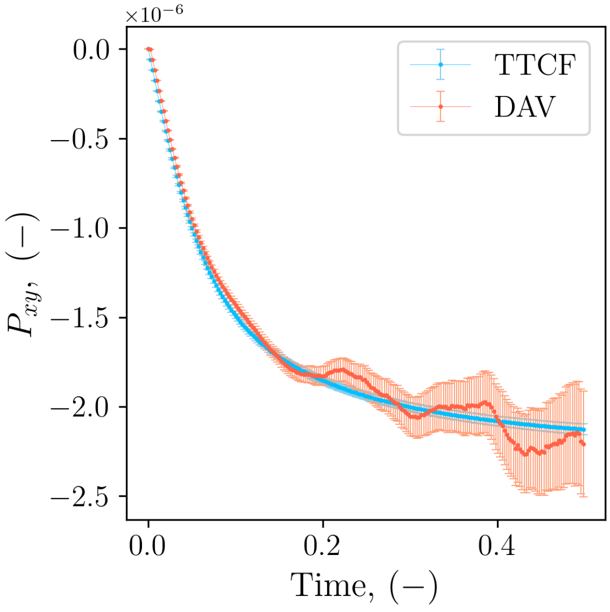

(b)

Figure S1: Evolution of P_{yx} at $\dot{\gamma} = 10^{-6}$ obtained using the modified LAMMPS version proposed by Sanderson and Searles [1]. a) Full simulation interval. b) Zoom of the initial time interval. Error bars represent the standard error. All quantities are reported in reduced units.

Some tests were performed for the DPD fluid. Results obtained with the modified LAMMPS version are reported in Figure S2. Also in this case, improved consistency between TTCF and DAV is observed relative to the results obtained with the official LAMMPS release (Fig. 6 in the manuscript). The comparison is less sharp than for LJ–WCA because DAV for DPD is intrinsically noisier, even at $\dot{\gamma} = 10^{-2}$. Nevertheless, the effect of the modified implementation on the DAV–TTCF agreement remains evident.

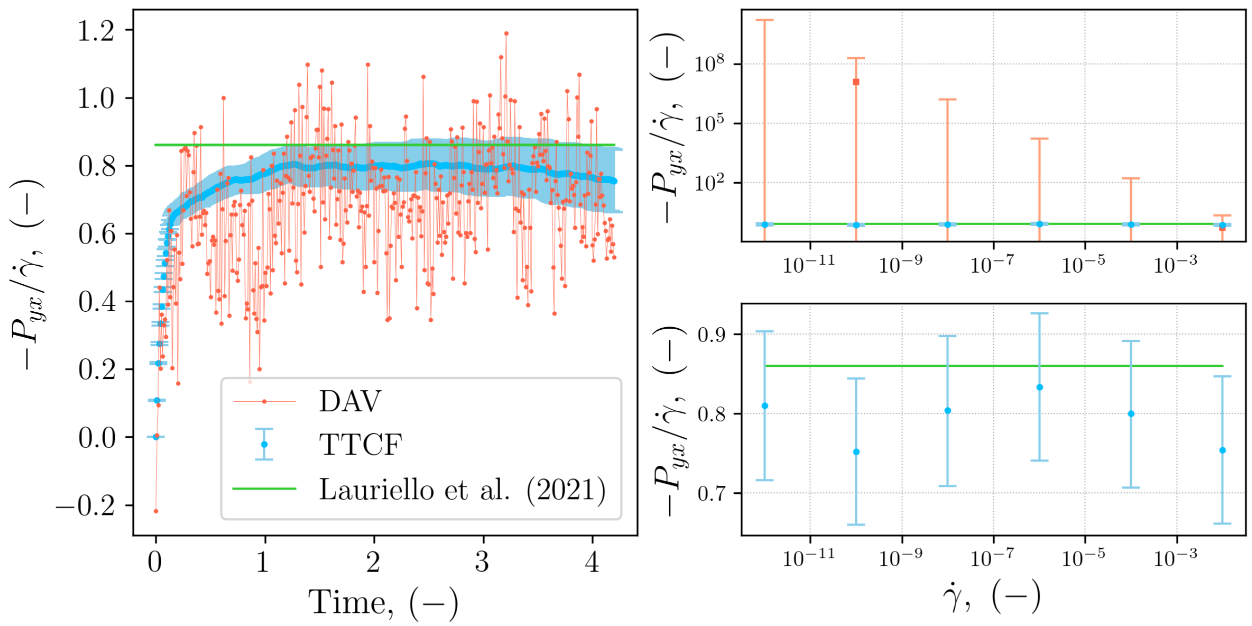

Figure S2: (left) Evolution of the shear viscosity of the DPD fluid at $\dot{\gamma} = 10^{-2}$ obtained with the modified LAMMPS version proposed by Sanderson and Searles[1]. (right) Shear viscosity of the DPD fluid at different shear rates obtained with the modified LAMMPS version. Error bars represent the 95% confidence interval. Values are in reduced units.

2 Comparison with previous results for LJ–WCA fluid

The TTCF viscosities reported in Figure 1 of Borzsák et al.[2] were extracted and compared with the results obtained here at the system. For clarity, Figure S3 reports the viscosity as a function of $\dot{\gamma}$, which directly reflects the shear-stress comparison at $t = 1.5$. The figure includes both the present results obtained with the official LAMMPS release and, for completeness, those obtained with the modified SLLOD implementation proposed by Sanderson and Searles[1]. Error bars represent the standard error for the present data, while an uncertainty of 4% is shown for Borzsák et al.[2], as reported in their work. Overall, the agreement is satisfactory within the combined uncertainties across the investigated shear rates. It is also noted that Borzsák et al.[2] employed a Gaussian thermostat, whereas a Nosé–Hoover thermostat is used here. This difference may contribute to minor shifts but does not affect the qualitative consistency of the TTCF results in the low shear rate regime considered.

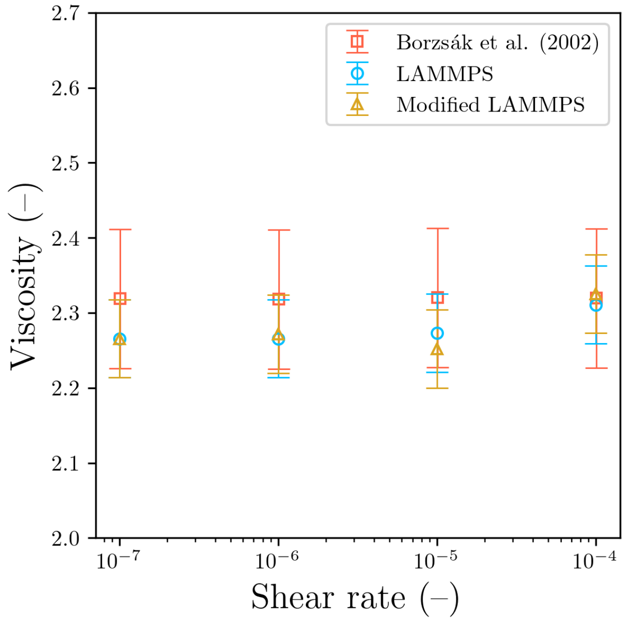

Figure S3: Comparison between the shear viscosity calculated in this work and the one reported by Borzsák et al.[2] for the LJ fluid at different shear rates. All results are obtained with TTCF with mappings. Error bars represent the standard error for the present work, while the uncertainty shown for Borzsák et al.[2] is 4%, as reported therein.

3 Comparison with Green–Kubo

While TTCF is primarily intended for shear-dependent rheology, a comparison with equilibrium methods can provide useful context for the present Newtonian validation case. Here, TTCF is compared against a Green–Kubo (GK) calculation performed for the same simple DPD fluid. The GK viscosity is computed from the stress autocorrelation function:

$$\mu = \frac{V}{k_B T} \int_0^\infty \langle P_{yx}(t_0)\, P_{yx}(t_0 + t) \rangle \, \mathrm{d}t, \tag{S-1}$$

which corresponds to the zero shear limit of the TTCF expression. For TTCF, 10^5 daughter trajectories are propagated for 420 time steps each, with time step $\Delta t = 0.01$, as described in the manuscript. For GK, the stress autocorrelation is evaluated from a single equilibrium trajectory using the LAMMPS `fix ave/correlate` with `Nevery`=1, `Nrepeat`=420, and `Nfreq`=420. The viscosity is then obtained by integrating the resulting autocorrelation

according to Equation (S-1). With this setup, the correlation function is accumulated over 420 lag times for each origin, and 10^5 correlation windows are sampled over a total simulation length of 420×10^5 time steps. It is worth noting that, even with this attempt to match the sampling effort, the two approaches remain intrinsically different: TTCF estimates are obtained from ensembles of short driven trajectories, whereas GK relies on an equilibrium autocorrelation accumulated and time averaged along a single long trajectory, so the time integral in Equation (S-1) is effectively built from averages constructed in a different manner. For the present setup, TTCF yields $\mu = 0.79 \pm 0.05$ (–), whereas GK yields $\mu = 0.84 \pm 0.01$ (–). The computational cost is also lower for GK: the GK calculation requires 6.6 core hours, whereas TTCF requires 63.3 core hours (core hours computed as number of CPU cores times wall time). As expected, equilibrium Green–Kubo is the most efficient choice for Newtonian fluids, whereas TTCF becomes essential when addressing shear-dependent response in non-linear regimes where equilibrium methods are not applicable.

References

(1) Sanderson, S.; Searles, D. J. On the importance of numerical integration details for homogeneous flow simulation. *The Journal of Chemical Physics* **2026**, *164*, 084121.

(2) Borzsák, István.; Cummings, P. T.; Evans, D. J. Shear Viscosity of a Simple Fluid over a Wide Range of Strain Rates. *Molecular Physics* **2002**, *100*, 2735–2738.
